# Identification of Metastasis Associated Antigen 1 (MTA1) by Serological Screening of Prostate Cancer cDNA Libraries

**DOI:** 10.2174/1874091X00802010100

**Published:** 2008-07-02

**Authors:** Geng Li, Deepak P Assudani, Aija Line, Fuming Cao, Amanda Miles, Robert C Rees, Stephanie E.B McArdle

**Affiliations:** 1 School of Science and Technology, Nottingham Trent University, Clifton Lane, Nottingham, NG11 8NS, UK; 2 Biomedical Research and Study Centre, University of Latvia, Latvia, LV-1067

**Keywords:** Tumour antigen, SEREX, EST and MTA1

## Abstract

Over the past 10 years the serological analysis of recombinant cDNA expression libraries (SEREX) has proved to be an effective method for the identification of tumour antigens. In the present study, two prostate cancer libraries were constructed and screened using autologous sera. Fifty five genes were isolated, including 46 known genes and 9 previously uncharacterised genes. Among the known genes, a metastasis-associated gene, MTA1, previously identified by differential cDNA hybridisation, was preferentially expressed in a panel of malignant tissues compared with normal tissues, as analysed by reverse transcriptase-polymerase chain reaction (RT-PCR). MTA1 transcripts were observed to be over-expressed in normal human testes as well as various cancer tissues when compared to the panel of normal tissues. MTA1 antigen reacted with 2 of 13 allogeneic prostate cancer patient sera tested, but no sera reactivity was observed to any of the normal adult sera tested. Furthermore, a similar distribution and expression level of MTA-1 was observed in murine tissues and cancer cell lines. Based on these findings and previous reports on the literature on this gene, MTA-1 can be considered not only as a “biomarker” of aggressive disease but also as a potential therapeutic target.

## INTRODUCTION

Identification of immunogenic cancer-associated proteins is a pre-requisite for the development of immunotherapy for cancer patients. The growing list of human tumour antigens recognised by cytotoxic T lymphocytes (CTLs) [1, 2] and antibodies [3, 4] provides convincing evidence of immune recognition by cancer patients and many are attractive targets for vaccine-based cancer therapies. Sahin and his colleagues developed a novel serological approach for the analysis

of tumour antigens by recombinant expression cloning (SEREX), identifying human tumour antigens able to elicit a humoral immune response. This strategy is based on the construction of cDNA expression libraries from tumour specimens and immunoscreening the libraries with cancer patient’s serum, thus allowing a systematic search for immunoreactive proteins. SEREX has been applied to a wide range of tumour types, “novel” as well as previously defined tumour antigens have been identified using SEREX, including MAGE-1 and tyrosinase, originally identified as tumour antigens recognised by CTLs [1, 2].

To date, over 2700 antigens have been submitted to the SEREX database (http://ludwig-sun5.unil.ch/CancerImmun-omeDB/SEREX_Intro.php). Although the majority of SEREX-defined genes have not been characterised beyond preliminary sequence analysis and determination of expres-

These authors contributed equally to the manuscript.

sion pattern, several have been proposed as attractive candi- dates for the construction of therapeutic cancer vaccines. For example, the cancer/testis antigen NY-ESO-1 was identified by screening an oesophageal cancer cDNA library and is regarded as one of most antigenic tumour antigens; antibody responses to NY-ESO-1 have been observed in 40-50% of patients with NY-ESO-1 expressing tumours and the presence of antibody strongly correlating with a CD8^+^ T-cell response to the antigen [5]. Clinical trials, investigating the immunological and clinical response to vaccination with NY-ESO-1 peptides, especially in melanoma, are ongoing [6].

In the present study we applied SEREX to identify prostate cancer-associated antigens by screening cDNA expression libraries constructed from prostate cancer tissue. Following immunoscreening of two prostate cancer cDNA expression libraries with autologous serum, a panel of immuno-reactive antigens were identified. Included in this panel was metastasis-associated antigen-1 (MTA1), a previously known antigen which we now report to be widely over-expressed in human and mouse tumours. Our results demonstrate that high levels of MTA1 transcripts are expressed in all murine tumour cell lines tested, compared with the low levels expressed in normal mouse tissues. MTA1 expression has previously been correlated with more aggressive tumours and may play a role in metastasis and invasion [7]. Moreover, because of its highly conserved sequence in humans and mice (96%), MTA1 may provide an ideal antigen to be studied as a ‘self-antigen’ in mouse tumour models with significant relevance for human cancer immunotherapy.

## MATERIALS AND METHODS

### Human Tissue Specimens, Tumour Cell Lines and Patient Sera

Tissue samples were obtained at the time of the surgery from the City hospital, Nottingham and Queen’s Medical Centre, Nottingham. All tumour tissue samples were verified histologically by observation of tissue sections by a clinical pathologist. Blood samples were obtained from patients and healthy male volunteers with no known evidence of benign or malignant prostatic disease and the serum collected was stored at -80°C. All patients gave informed consent and this study received local ethical approval. PC3 and DU-145 human prostate carcinoma cell lines were maintainedin Dulbecco's modified Eagle's medium supplemented with 10% heatinactivated foetal calf serum (FCS); the LNCaP human prostate carcinoma cell line was cultured in RPMI 1640 medium with 10% FCS, 2mM L-glutamine, testosterone (5ng/ml) and hydrocortisone (5ng/ml).

### Murine Cell Lines and Tissues

CT26 (murine colon carcinoma) cells were maintained in DMEM media supplemented with 2mM L-glutamine and 10% FCS; B16-F1 (murine melanoma) and CMT 93 (murine rectal carcinoma) cells, A20 (B cell lymphoma) cells and RENCA, a BALB/c renal carcinoma cell line were maintained by serial *in vitro* passage in RPMI 1640 tissue culturemedium supplemented with 2 mM L-glutamine, 10% FCS, and 0.05mM 2-ME.Normal mouse tissues were harvested from naïve Balb/c mice and were immediately snap frozen in liquid nitrogen and stored at -80˚ C.

### Construction of cDNA Expression Libraries

Two cDNA expression libraries were constructed from two tumour specimens of a well/moderately differentiated prostatic adenocarcinoma and an undifferentiated prostatic adenocarcinoma, respectively. These two specimens were obtained from Professor F Hamdy from patients attending Newcastle University under local ethical approval. Total RNA was isolated using RNA STAT-60 (ams Biotechnology, UK) according to the manufacturer’s protocol. Poly(A)^+^ RNA was purified from total RNA using a mRNA isolation kit (Stratagene UK) and cDNA was ligated into the ZAP Expression vector using Gigapack III Gold cloning kit (Stratagene UK). After* in vitro* packaging, two libraries containing 1.2(10^6^ and 2.0(10^6^ primary cDNA clones respectively were obtained.

### Immunoscreening of the cDNA Library

Immunoscreening of the cDNA library was performed as described by Sahin *et al*. [3]. Briefly, 1:10 diluted autologous patient’s serum and allogeneic sera, were preabsorbed with *E.coli*-phage lysate to remove antibodies reactive with antigens related to the vector system. In order to eliminate cDNA clones encoding human immunoglobulins, membranes were pre-screened with AP-conjugated rabbit anti-human IgG, Fcγ fragment specific secondary antibody (Pierce, USA) prior to incubation with sera. Reactive plaques were detected with 5-bromo-4-chloro-3-indolyl-phosphate/ nitroblue tetrazolium (BCIP/NBT), marked and excluded from further study. The membranes were then incubated with pre-absorbed 1:200 diluted patient’s serum and serum-reactive clones were detected with AP-conjugated secondary antibody and visualized by incubating with BCIP/NBT. The reactive phage clones were subcloned to monoclonality and converted to pBK-CMV phagemids. To assess frequencies of antibody responses to the SEREX defined antigens in individual allogeneic sera from prostate cancer patients and normal individuals, *E. coli* were transfected with approximately equal numbers of positive phage and non-recombinant phage. These were then screened with 1:200 diluted individual allogeneic serum samples as described above, excluding the IgG pre-screening step.

### DNA Sequencing and Sequence Analysis

Phagemid DNA was purified using QIAprep Spin Miniprep kit (QIAGEN, West Sussex, UK), analysed by *EcoRI/XhoI* restriction enzyme digestion and clones representing different cDNA inserts were sequenced using an ABI PRISM 310 automatic sequencer (Applied Biosystems UK). Sequence alignments were performed using the GenBank database (www.ncbi.nlm.nih.gov). Genes identical with entries in the GenBank were classified as known genes, whereas those that shared sequence similarity only with ESTs and those that had no similarity to entries in either the GenBank or EST databases were classified as unknown genes. Multiple sequence alignments were performed with DNASIS (Hitachi Software Engineering Co Ltd) and MACAW (NCBI) software. Chromosomal localisation and exon-intron organisation for uncharacterised cDNAs was determined by comparison to the working draft of the human genome.

### Conventional Reverse Transcription PCR (RT-PCR) Analysis

To evaluate the mRNA distribution of SEREX-defined antigens, total RNA from a panel of normal human tissues (lung, liver, brain, trachea, heart, and kidney) was purchased from Clontech, (UK). Total RNA from normal testes, oesophageal cancer and adjacent non-tumour tissues, head and neck tumour and adjacent non-tumour tissues, prostate cancer tissues, stomach cancer and kidney cancer tissues and cell lines (DU-145, LNCaP and PC3) was isolated using RNA STAT-60 reagent (ams Biotechnology, UK) according to the manufacturer’s protocol. For mouse tissue screening, several tissues were dissected from mice and immediately snap frozen. Tissues were then ground in liquid nitrogen before proceeding to total RNA isolation using RNA STAT-60. The first-strand cDNA was synthesised from 2μg of total RNA primed with oligo-dT(15) (Promega UK) as described in the manufacturer’s protocol. Human MTA1 (5’-CGCTCAAGTCCTACCTGGAG and 3’-TGGTACCGGT TTCCTACTCG) and GAPDH (5’-ACCACCAACTGCTTA GCACC and 3’-CCATCCACAGTCTTCTGGGT) specific primers were used to verify expression of hMTA1. The mouse MTA1 gene specific primers (5’-GCGAGAGCTGT TACACCACA and 3’-ACTGCTGAGCACACTGGATG) and murine GAPDH primers (5’-GGTGAAGGTCGGAGT CAACGGA and 3’-GAGGGATCTCGCTCCTGGAAGA) were designed with the assistance of the Primer3 website and obtained lyophilized from Sigma Genosys (UK). The gene specific PCR primers were designed to ensure that the primer sequences were located within different exons and that the PCR products obtained were 300-500bp in length. GAPDH was used as an internal control to observe the relative amounts of cDNA from each sample that was used in the RT-PCR reaction. The cDNA templates were used in the PCR in a final volume of 50 µl containing 0.4 μM of each primer, 1.5 mM MgCl_2_, 0.2 mM deoxynucleotide triphosphates (dNTPs) and 2.5 U Taq polymerase (Promega, UK).

PCR conditions were as follows for MTA1: Initial denaturation at 95ºC for 5 mins, followed by 34 cycles of denaturation at 95ºC for 1 min, annealing at 58ºC for 1 min and extension at 72°C for 1 min. This was followed by a final extension step at 72°C for 5 mins. GAPDH PCR conditions consisted of 25 cycles of denaturation at 94ºC for 45 secs, annealing at 58ºC for 45 secs and extension at 72°C for 45 secs. Semi-quantitative analysis of RT-PCR products was performed by 1.5 % (w/v) agarose gel electrophoresis and ethidium bromide visualization. After normalization to GAPDH mRNA as a control for each cDNA template, the expression of gene-specific mRNA in the cDNA samples tested was compared.

## RESULTS

### Identification and Characterisation of Immunoreactive cDNA Expression Clones

After immunoscreening of 8 x 10^5^ pfu from each of two prostate carcinoma cDNA expression libraries with the autologous serum, a total of 26 and 73 immunoreactive clones were identified from first and second cDNA expression libraries, respectively. These clones were purified, *in vivo *excised, and converted to pBK-CMV plasmid forms. The cDNA inserts were analysed using restriction mapping and sequencing. Sequence comparison against GenBank and EST databases showed that these clones were derived from 54 distinct genes, which were designated as Pr1-Pr54. Pr1-Pr11 were derived from the first cDNA library and the remaining clones from the second library. Eight genes showed no sequence similarity or only partial sequence similarity with genes in the GenBank database but shared sequence similarity with ESTs derived from various tissues (referred to as “unknown genes”) (Table **1**).

We therefore investigated these genes further and EST analysis of Pr3 (BC017874), Pr9 (BC062419), Pr10 (XM_496394), Pr11 (AC011375), Pr43 (NM_014929) and Pr50 (BC007379), showed that they shared sequence identity with reported ESTs derived from various normal tissues, *e.g.* lung, liver, kidney, brain and B cells, suggesting that these genes are widely expressed in human tissues (Table **1**). Pr6 (AC104841) transcripts on the other hand looked more promising as no similarities with normal tissue were found which was confirmed in a panel of RNAs isolated from normal human tissues as determined by RT-PCR after 40 cycles of amplification. Unfortunately we were also unable to detect it in a panel of tumour tissues or tumour cell lines. The last one, Pr52, is located on chromosome 7p21 and is similar only to an EST derived from human placenta (DA832074). The tissue expression pattern of this gene was therefore evaluated by RT-PCR and results showed that Pr52 was expressed in normal brain, kidney, thyroid, testis and placenta (data not shown). It was however not found at the time of this investigation whether the expression of these genes was higher in cancer tissues compared to normal tissue. Further work is now being carried out to evaluate this using real-time RT-PCR.

The remaining 46 genes showed sequences identical with or highly similar to known GenBank entries (“known genes”) (Table **2**). Three of these had been previously isolated from other tumour tissues by SEREX (Pr 17, 48 and 54). Clone Pr54 is identical to human transcriptional coactivator tubedown-100 (TBDN100), which is involved in regulation of angiogenesis and was isolated by us from gastric cancer and is overexpressed in gastric cancer tissues compared with adjacent non-cancer tissues (Ga19, GenBank accession number AY039242) [4]. Clone Pr48 is similar to the gene encoding human ankyrin repeat domain 17, which has also been isolated from breast cancer (NY-BR-16, Genbank accession number AF308285) [8]. Clone Pr17 is identical to human NEDD8 ultimate buster-1 (NUB1) mRNA, which was detected in renal cell carcinoma (NY-REN-18, Genbank accession number AF155099) [9].

### Immune Recognition of the SEREX-Defined Antigens by Serum Antibodies from Prostate Cancer Patients and Normal Individuals

To determine whether immune recognition of antigens encoded by SEREX genes was tumour-related, allogeneic sera from normal individuals and patients with prostate can-

cer were tested for antibody reactivity against selected antigens using a protein array system. Of the 35 antigens screened, 17 antigens reacted with a subset of sera from both normal individuals and cancer patients; thus, approximately half (17/35) of the antigens identified in this study were non-cancer related and were classified as naturally occurring autoantigens. Nine proteins had a cancer-related serological profile, reacting only or mainly with sera from prostate cancer patients. The remaining antigens only reacted with autologous serum and not with any allogeneic sera from normal individuals or patients with prostate cancer (Table **3**).

From the panel of genes identified, we decided to focus on genes which did not generate any reactivity to serum obtained from normal patients, since these are more likely to demonstrate cancer-specific immunological responses. These genes included the aarF domain containing kinase 4 (ADCK4), for which very little information could be found; the ribosomal protein S10, which has previously been shown to be deregulated in colorectal cancer samples, along with larger transcripts being produced in addition [10]; the cell death regulatory protein GRIM19, which appears to be an important cell death regulatory protein and is involved in mitochondrial metabolism. Loss of GRIM-19 expression has been shown to be associated with renal cell carcinomas. Interestingly, GRIM-19 mutations have been observed in mitochondrial rich tumours of thyroid [11]. Considering that it is one of the critical regulatory proteins and its expression is responsible for tumour cell death, it seems an unlikely antigen for immunotherapy. And finally, MTA1 which was found to represent the most promising gene based on previously published literature reporting on its overexpression in various cancers. Moreover, being highly expressed in metastatic cells suggests that it would be a highly specific therapeutic target which could be potentially used to prevent disease relapse in advanced disease states. Also, several studies have clearly established that MTA1 overexpression leads to cell transformation and by suppressing MTA1 expression you get an inhibition of cell growth [12,13].

**Fig. (1):**
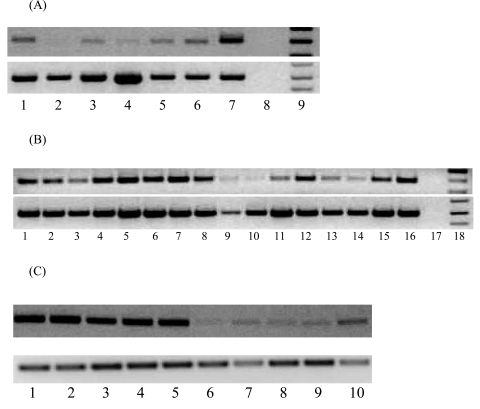
**(A)** Expression of MTA1 in normal human tissues analysed by RT-PCR. Cycling conditions were optimized so that the RT-PCR products were analyzed when amplification is within the linear phase. Lanes 1-9 from left to right, normal brain, kidney, heart, liver, lung, PBMC, testis, negative control and DNA ladder. **(B)** Tissue distribution of MTA1 transcript in malignant tissues and cell lines was analysed by RT-PCR. Lanes 1-4: prostate cancer tissues; lanes 5-7: prostate cancer cell lines (lane 5: DU-145; lane 6: LNCaP; lane 7: PC3); lanes 8-11: renal cancer; lanes 12-16: gastric cancer. Amplification of GAPDH was also performed in parallel as a control in all samples. **(C)** Agarose gel electrophoresis of mGAPDH and mMTA1 following RT- PCR from murine tumour cell lines and tissues. Lanes 1-10 from left to right, CT26, A20, RENCA, CMT, B16, liver, lung, muscle, spleen, kidney.

The expression pattern of MTA1 transcripts in a panel of normal human tissues, matched human non-tumour and tumour tissues, human prostate cancer tissues and cell lines was analysed by RT-PCR. Weak expression of MTA1 transcript was detected in normal human tissues except testis, where MTA1 was highly expressed (Fig. **1A**). Human head and neck tumours and oesophageal cancer tissues expressed higher levels of MTA1 transcripts than matched non-tumour tissues (data not shown). MTA1 mRNA is highly expressed in human prostate cancer cell lines and a significant number of prostate, renal and stomach cancer tissues (Fig. **1B**). We further analysed the expression of MTA1 in murine tissues and tumour cell lines since MTA1 is highly conserved in humans and mouse demonstrating 96% similarity at the protein level. MTA1 was found to be over-expressed in all murine tumour cell lines as compared to normal tissues, except the testis (Fig. **1C**), which showed a similar level of expression to tumour tissues (data not shown). In comparison, all the normal tissues examined expressed MTA1 at very low levels (Fig. **1C**). Interestingly, we found that MTA1 was expressed at low levels in muscle tissue compared to other tissues, which contradict previously published work where no MTA1 expression was found [14].

## DISCUSSION

A number of autoimmunogenic tumour antigens have been identified by screening with autologous CTL. However, the methodology for defining T cell-recognised tumour antigens is dependant on generating T cell clones and establishing tumour cell lines; conditions that are frequently difficult to meet and virtually impossible in the case of certain tumour types, such as prostate cancer. In the present study, using a serological technique termed SEREX, we have identified 54 different antigens by immunoscreening two cDNA expression libraries constructed from two different prostate cancer tissues using autologous serum. Eight antigens were encoded by unknown genes and the remaining 46 were identified from database screening, three having been previously detected by SEREX analysis in other tumour types – Pr17 in renal cell carcinoma, Pr48 in breast cancer and Pr54 in gastric cancer. Our data has further demonstrated that SEREX is an efficient technique for the identification of tumour antigens that elicit humoral immune response in cancer patients.

To determine which of our panel of antigens showed a tumour-restricted immunogenicity, we analysed the frequency of the antibody responses using sera from cancer patients as well as healthy individuals. Nine antigens reacted with sera from allogeneic prostate cancer patients (in addition to autologous serum reactivity), indicating that the immune response to these antigens is cancer-associated. Three of these antigens are encoded by unknown genes (Pr3, Pr6 and Pr Pr52 respectively); additionally three of them (Pr8-ribosomal protein L27a, Pr16-MTA1 and Pr31-ribosomal protein S10) have previously been reported to be over-expressed in cancer [10, 15]. Screening, using a larger panel of sera from patients with other cancers is required to establish the cancer specific immunogenicity of these antigens.

This study has identified a number of immunogenic self–proteins demonstrating sera–reactivity with both normal and prostate cancer patients sera. A possible reason for the immunogenicity of self-proteins are structural changes resulting from mutations, translocations or splice variants. We have identified previously unknown splice variants of two genes, geminin, a DNA replication inhibitor represented by clone Pr14, and HRC1, a DNA-binding protein represented by clone Pr13 (unpublished data). Alterations in the pattern and efficiency of alternative splicing of several pre-mRNAs (e.g. CD44, BRCA1, WT-1) have been implicated in tumourigenesis and correlated with tumour progression [16, 17]. De-regulation of splice site selection may serve as an additional mechanism for the generation of protein diversity contributing to the selection of more aggressive tumour cells [18,19]. Interestingly, alternate splice variants for the murine MTA1 gene have recently been discovered in various mouse tissues and these variants were differentially localised within the cell [20]. However, their functional significance is not yet known and it would be of interest to determine whether human tissues express similar splice variants.

Several SEREX antigens identified in this study have been previously reported to be upregulated in different types of cancer; HRC1 (Pr12) was reported to be overexpressed in the bladder carcinoma cell line [21]; programmed cell death 6 (Pr15) was found to be directly involved in apoptosis and was upregulated in rat liver hepatoma and human lung cancer [22]; MTA1 (Pr16) was originally identified by differential cDNA hybridisation using RNA from metastatic and non-metastatic rat mammary adenocarcinoma cell lines [23]; human MTA1 was initially cloned by homology searching to rat MTA1 [24] and is a component of the histone deacetylase and nucleosome-remodelling complexes.

In the current study, MTA1 has been shown for the first time, to elicit a humoral immune response in prostate cancer patients. The expression pattern of MTA1 in normal and tumour tissues was analysed by RT-PCR and the data extends previously published findings, where MTA1 was shown to be over-expressed in prostate cancer cell lines, prostate cancer, renal cancer, gastric cancer, oesophageal cancer, and head and neck tumour tissues but only weakly expressed in normal tissues except the testis. We also analysed the expression pattern of the murine MTA1 gene in different tissues and in tumour cell lines. Most of the tissues expressed MTA1 transcripts at low levels, whereas high levels of expression were observed in all tumour cell lines. These results have been confirmed by real time RT-PCR and western blotting (unpublished results). Several peptides, MHC class-I and class-II, derived from MTA1 were identified using the online-based algorithm “SYFPEITHI” and synthesised. Using our transgenic mouse model, many of them were able to induce the generation of cytotoxic T lymphocytes that were capable of killing peptide-pulsed target cells but none of them could kill tumour cells naturally over-expressing MTA1 suggesting that these peptides were not endogenously processed. These were also confirmed after immunisation with cDNA encoding for the whole of MTA1 gene (data not shown).

In conclusion, we screened two prostate cancer libraries using autologous sera and isolated 54 genes, including 46 known genes and 8 previously uncharacterised genes. Among these, we identified MTA1 and our data extends previously published studies regarding its over-expression in aggressive tumours. MTA1 has previously been found to be associatedwith progression to the metastatic state in various cancers and our observations suggest that MTA1 may be a useful target for immunotherapy of cancer in the advanced and metastatic stages of disease progression even though we have so far failed to find naturally endogenously processed MTA1 derived peptides. It is also possible that strong immune tolerance exists towards such ubiquitously expressed proteins and therefore a stronger means of immunisation would be required in order to break tolerance.

## Figures and Tables

**Table 1.** Novel Proteins Identified by SEREX Analysis of Prostate Cancer cDNA Expression Libraries with Autologous Sera

**Designation**	**No of Clones**	**GeneBank Acc. nos**	**Descriptive feature**
Pr3	2	BC017874	Hypothetical protein MGC40405. EST: lung, stomach, ovary, brain etc.
Pr6	3	AC104841	BAC clone RP11-475G3
Pr9	1	BC062419	Family with sequence similarity 36. EST: Lung, liver, skin, kidney etc.
Pr10	1	XM_496394	Similar to KIAA1693 protein. EST: Brain, liver, spleen, stomach, heart etc.
Pr11	1	AC011375	EST: Foetal pancreas, embryonic stem cells, pooled tumours etc.
Pr43	1	NM_014929	KIAA0971 protein. EST: liver, kidney, stomach, placenta, testis etc.
Pr50	1	BC007379	Hypothetical protein MGC16207. EST: carcinoma, neuroblastoma, lung, colon etc.
Pr52	1	AC004982	PAC clone RP5-1159O4 from 7p21-p22. EST: only similar to an EST derived from the placenta

**Table 2.** Known Proteins Identified by SEREX Analysis of Prostate Cancer cDNA Expression Libraries Screened with Autologous Sera

**Designation**	**No of Clones**	**GeneBank Acc. nos**	**Identity**
Pr1	3	NM_002078	Trans-Golgi P230
Pr2	2	BC027473	aarF domain containing kinase 4
Pr4	1	NM_015265	SATB family member 2
Pr5	6	AY495330	Mitochondrial protein
Pr7	3	NM_001012	Ribosomal protein S8
Pr8	1	NM_000990	Ribosomal protein L27a
Pr12	6	M91083	DNA-binding protein (HRC1)
Pr13	1	M91083	A new spliced isoform of HRC1
Pr14	7	NM_015895	A new spliced isoform of geminin, DNA replication inhibitor
Pr15	1	NM_013232	Programmed cell death 6.
Pr16	1	NM_004689	Metastasis associated 1
Pr17	5	AF155099	NY-REN-18 antigen ; NUB1
Pr18	1	NM_006753	Surfeit 6
Pr19	1	M34667	Phospholipase C-gamma
Pr20	1	NM_004501	Scaffold attachment factor A
Pr21	1	NM_003367	Upstream transcription factor 2
Pr22	1	NM_005154	Ubiquitin specific protease 8
Pr23	2	BC006398	Vesicle docking protein p115
Pr24	3	NM_002792	Proteasome (PSMA7)
Pr25	1	NM_203380	Acyl-CoA synthetase long-chain family member 5 (ACSL5)
Pr26	1	NM_006356	ATP synthase
Pr27	2	BC058921	Glutamyl-prolyl-tRNA synthetase
Pr28	1	NM_006013	Ribosomal protein L10
Pr29	1	BC020515	Ribosomal protein S14
Pr30	1	NM_000980	Ribosomal protein L18a
Pr31	1	NM_001014	Ribosomal protein S10
Pr32	1	NM_000994	Ribosomal protein L32
Pr33	1	BC000443	Tumor protein p53 inducible protein 11.
Pr34	4	BC037545	Poly (ADP-ribose) polymerase family, member 1
Pr35	1	NM_003747	Tankyrase
Pr36	1	HSA010840	ATP-dependent RNA helicase
Pr37	1	NM_006201	PCTAIRE protein kinase 1
Pr38	1	NM_001694	ATPase, H+ transporting
Pr39	1	NM_005627	Serum/glucocorticoid regulated kinase
Pr40	3	NM_002032	Ferritin, heavy polypeptide 1
Pr41	1	NM_015965	Cell death-regulatory protein GRIM19
Pr42	1	BC019315	N-acetylneuraminic acid synthase
Pr44	6	AF413079	Centaurin gamma3
Pr45	2	AF438201	Tankyrase II
Pr46	1	BC070234	Ankyrin repeat domain 12
Pr47	1	NM_012111	AHA1, activator of heat shock 90kDa protein ATPase homolog 1
Pr48	2	AF308285	NY-BR-16; ankyrin repeat domain 17
Pr49	1	NM_016505	Putative S1 RNA binding domain protein
Pr51	1	NM_173593	Beta 1,4-N- acetylgalactosaminyltransferase- transferase-III
Pr53	1	NM_133636	DNA helicase HEL308
Pr54	1	AY039242	Gastric cancer antigen Ga19

**Table 3.** Reactivity of SEREX-Defined Antigens Screened with Sera from Healthy Donors and Patients with Prostate Cancer

Designation	Identity	Serological reactivity	
		Control	Pca
Pr1	Trans-Golgi P230 autoantigen	5-May	9-Sep
Pr2	aarF domain containing kinase 4	0/14	4/13
Pr3	Unknown	2/14	6/7
Pr4	SATB2	1/14	2/13
Pr5	Mitochondrical protein	0/4	0/9
Pr6	Unknown	3/13	12/14
Pr7	Ribosomal protein S8	6/13	13/14
Pr8	Ribosomal protein L27a	0/13	1/14
Pr9	Hypothetical protein LOC116228	1/10	1/7
Pr10	Similar to AB25	2/13	6/14
Pr11	Unknown	0/7	0/6
Pr12	DNA-binding protein (HRC1)	0/13	0/8
Pr13	A alternatively spliced isoform of HRC1	0/13	1/8
Pr14	Geminin, DNA replication inhibitor	1/13	1/8
Pr15	Calcium binding protein (ALG-2)	1/17	4/13
Pr16	Metastasis-associated1 (MTA1) mRNA	0/13	2/13
Pr17	NEDD8 ultimate buster-1	1/17	1/13
Pr18	Surfeit 6	4/13	3/8
Pr19	Phospholipase C-gamma mRNA	0/13	0/8
Pr20	Scaffold attachment factor A	0/13	0/8
Pr21	Upstream transcription factor 2, c-fos interacting	0/13	0/8
Pr22	Ubiquitin specific protease 8	3/17	7/13
Pr23	Vesicle docking protein p115	0/13	0/8
Pr24	Proteasome subunit HSPC	6/17	10/13
Pr25	fatty-acid-Coenzyme A ligase, long-chain 5	4/17	8/13
Pr26	F1F0-type ATP synthase subunit d mRNA	0/13	0/8
Pr27	glutamyl-prolyl-tRNA synthetase	5/17	10/13
Pr28	Ribosimal protein L10	2/6	5/10
Pr29	Ribosomal protein S14	2/5	5/10
Pr30	Ribosomal protein L18a	3/5	4/10
Pr31	Ribosomal protein S10	0/4	5/10
Pr32	Ribosomal protein L32	0/4	0/10
Pr35	Tankyrase	2/4	4/10
Pr41	Cell death-regulatory protein GRIM19	0/6	5/12
Pr52	Unknown	2/11	13-Nov
